# Case Report: Botulinum Toxin-A for Complication of Exposure Keratopathy Following Frontalis-Orbicularis Oculi Muscle Flap Shortening

**DOI:** 10.3389/fmed.2022.877162

**Published:** 2022-04-13

**Authors:** Chun-Chieh Lai, Chia-Chen Lin

**Affiliations:** ^1^Institute of Clinical Medicine, College of Medicine, National Cheng Kung University, Tainan, Taiwan; ^2^Department of Ophthalmology, National Cheng Kung University Hospital, College of Medicine, National Cheng Kung University, Tainan, Taiwan

**Keywords:** blepharoptosis, botulinum toxin-A, exposure keratopathy, frontalis-orbicularis oculi muscle flap, lagophthalmos

## Abstract

**Background:**

Lagophthalmos with exposure keratopathy is a potential vision-threatening complication following surgery for blepharoptosis. We report three cases successfully treated with botulinum toxin-A (Botox^®^, Allergan, Irvine, USA) for this complication.

**Cases:**

Three patients presented with severe blepharoptosis after surgery for orbital and frontal base tumors. They obtained good appearances after frontalis-orbicularis oculi muscle (FOOM) flap shortening. However, exposure keratopathy developed after the surgery despite frequent use of topical lubricants and autologous serum eye drops. We injected 5-10 units of botulinum toxin-A around the central supra-brow area, which was near the origin of the FOOM flap. One week later, they developed ptosis and could close the eye completely. The corneal defect gradually resolved. They recovered from ptosis 3 months later and never required a second injection.

**Observations:**

Lagophthalmos with exposure keratopathy is a potential vision-threatening complication following FOOM flap surgery. In severe cases, surgical revision should be considered to partially or totally release the FOOM flap attachment, which also decreases its function permanently. In this case series, we demonstrated that injecting botulinum toxin-A may be a promising method to manage this complication without permanently affecting the function of the FOOM flap.

**Conclusions:**

A botulinum toxin-A injection may be an effective treatment for patients developing exposure keratopathy after FOOM flap surgery.

## Introduction

Blepharoptosis is a complex disease with multiple etiologies, including previous orbital surgery and trauma, which are especially difficult to manage (1, 2). The choice of surgical technique depends largely on the levator muscle function (LMF) (3, 4). For fair to good LMF, the Müller's muscle-conjunctival resection, levator muscle advancement or resection may be adequate (5). A modification of the Putterman technique, the elevator muscle anterior resection, provides even better accuracy and reliability for ptosis correction (6). However, for patients with poor LMF, typically ≤ 4mm, supramaximal levator resection beyond Whitnall ligament has to be done. The technique is currently less performed for previous studies have shown that the upper lids became less elastic after large portions of the levator muscle were severed. Restrictions of downward saccadic movements, reduced blinking, and abnormal lid contour were reported postoperatively (7). The frontalis sling operation, a more popular and reliable surgical technique, relies on the recruitment of the ipsilateral frontalis muscle to lift the eyelid (8). Postoperative lagophthalmos, however, is a common complication. This technique is also complicated by the risks of infection and granuloma formation associated with the synthetic materials used for suspension, or the need of a second donor site if autologous materials are used (9).

**Figure 1:**
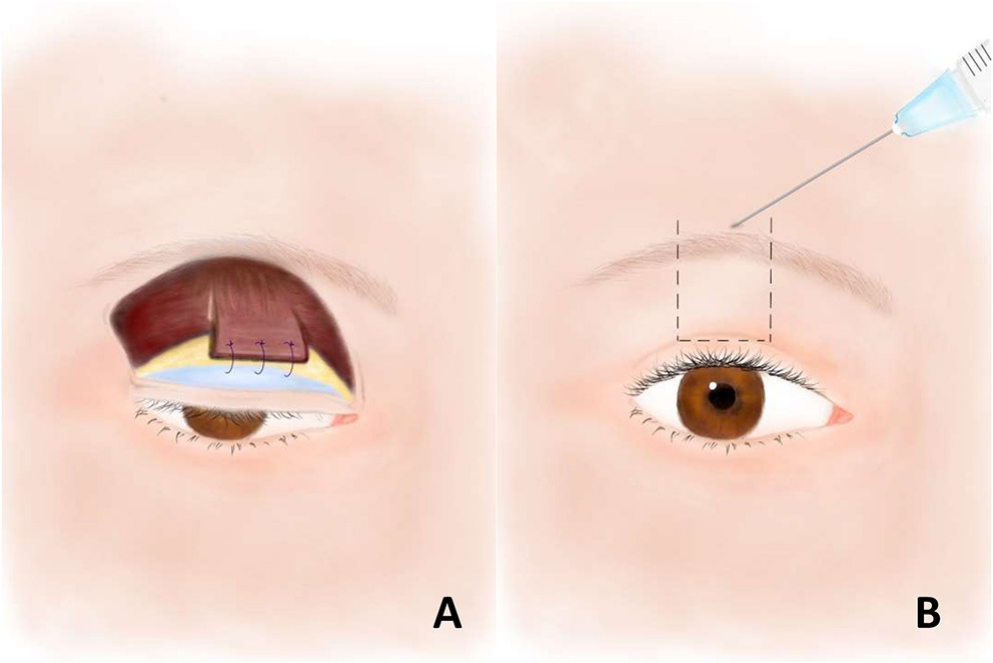
**(A)** The frontalis-orbicularis oculi muscle (FOOM) flap with the vertically oriented frontalis muscle at the upper part and the horizontally oriented orbicularis oculi muscle at the lower part. The flap is sutured to the upper tarsus after shortened to the desired length. **(B)** Botulinum toxin-A injection at the central supra-brow area, the origin of the FOOM flap. The area within the dashed lines represents the presumed location of the underlying FOOM flap.

In the 2000s, Lai et al. introduced the frontalis-orbicularis oculi muscle (FOOM) flap shortening technique to correct blepharoptosis with poor LMF (10). The technique is based on the finding that the vertically oriented frontalis muscle is continuous with the horizontally oriented orbicularis oculi muscle (11). The FOOM flap is harvested through dissection beneath the orbicular oculi muscle beyond the supraorbital rim (Figure 1A). After the desired position of the upper lid margin is decided, the local flap is anchored to the upper tarsus. The FOOM flap has demonstrated both contractility and elasticity, properties not possessed by a frontalis sling. This operation also spares the need of a second surgical site (12).

In this article, we present three cases who underwent FOOM flap shortening technique for blepharoptosis with poor LMF due to previous orbital surgeries. However, they developed lagophthalmos with exposure keratopathy, a possible vision-threatening complication following this surgery. The suspension power of FOOM flap is generated mainly from the frontalis muscle, and the orbicularis oculi muscle serves as a connector to elevate the ptotic eyelid. Therefore, if the power of frontalis muscle could be reduced, such as by injections of neurotoxic drug at the supra-brow area, the origin of the FOOM flap, lagophthalmos may be resolved (Figure 1B). Here, we report the effect of botulinum toxin-A (Botox^®^, Allergan, Irvine, U.S.A.) as a treatment for exposure keratopathy, a complication of FOOM flap surgery.

## Case Reports

### Case 1

**Figure 2:**
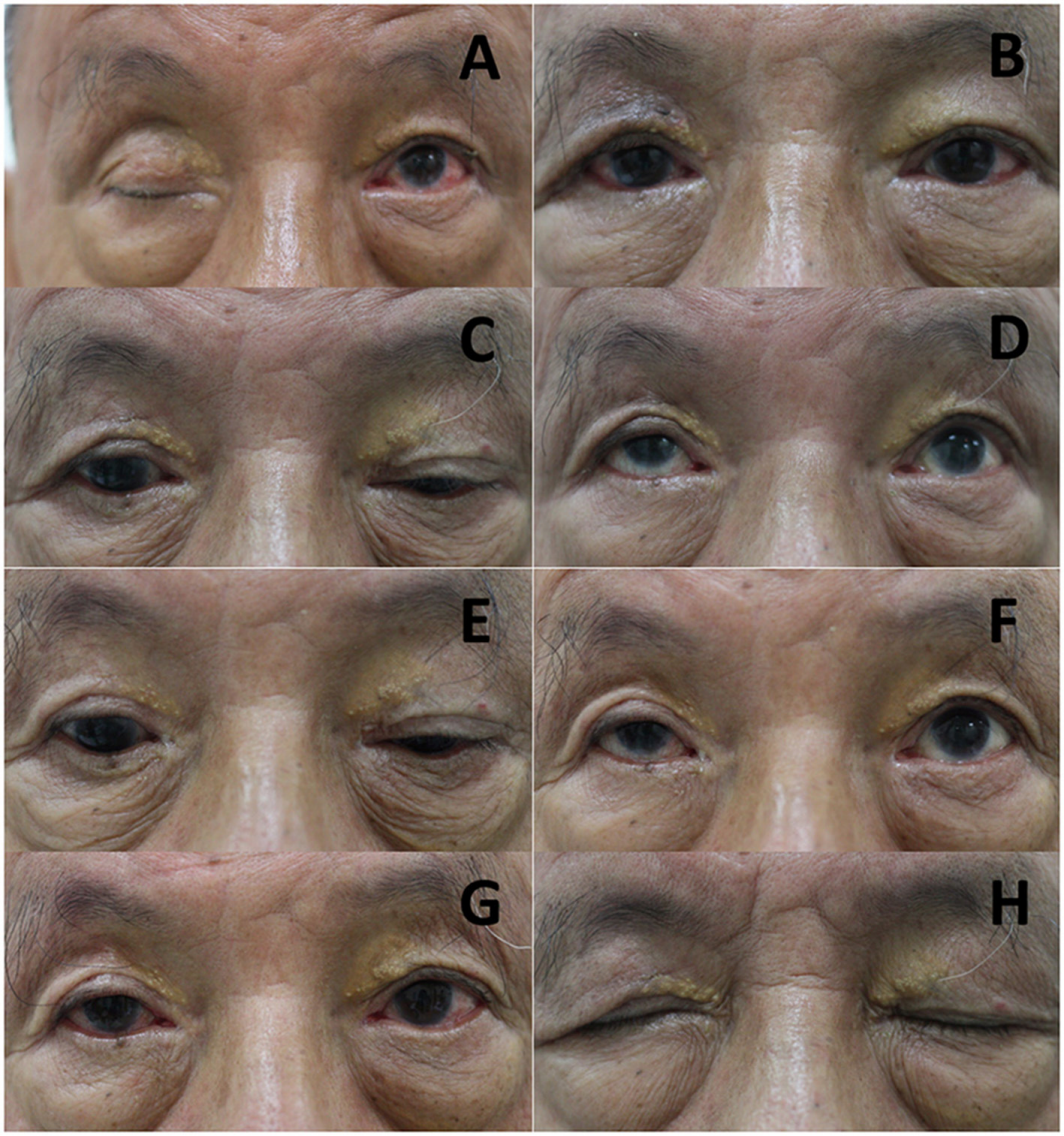
Serial photos of Case 1 before and after frontalis-orbicularis oculi muscle (FOOM) flap shortening surgery and injection of botulinum toxin-A. **(A)** Photos were taken before and **(B)** 3 months after FOOM flap shortening, respectively. **(C,D)** The FOOM flap function (upper eyelid excursion) of the right eye was about 4 mm 1 year after FOOM flap shortening. **(E,F)** The FOOM flap function was reduced to nearly 0 mm 1 week after the injection of botulinum toxin-A with 5 units at the central supra-brow area, which was supposed to be the area around the origin of the FOOM flap. **(G)** 1 week after the injection, he developed blepharoptosis. **(H)** The patient could close his eyes completely without lagophthalmos.

**Figure 3:**
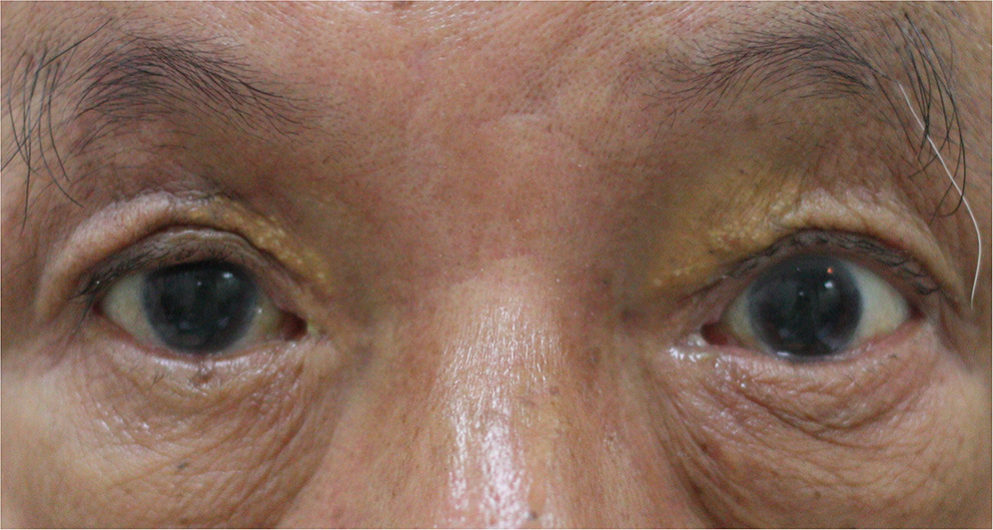
The blepharoptosis recovered with margin reflex distance 1 (MRD1) measured 2.5 mm 3 months after the injection.

A 63-year-old gentleman developed complete blepharoptosis for 6 months after surgery for an orbital lymphoma in the right eye (Figure 2A). We performed FOOM flap shortening, after which he obtained a symmetric appearance (Figure 2B). The margin reflex distance 1 (MRD1) was 2.5 mm, and the function of FOOM flap (upper eyelid excursion) was 4 mm postoperatively (Figures 2C,D). However, he developed lagophthalmos with exposure keratopathy following cataract surgery on the right eye, which was performed 1 year after. Due to the fact that there was a persistent corneal defect in spite of our best efforts with conservative treatments such as topical lubricants and autologous serum eye drops, we injected 5 units of botulinum toxin-A at the central supra-brow area. One week later, he developed blepharoptosis with MRD1 of 1 mm and the function of FOOM flap was reduced from 4 mm to nearly 0 (Figures 2E-H). He could close his right eye completely and experienced an improvement in his ocular surface. The corneal epithelial defect healed gradually. He recovered from the ptosis with MRD1 of 2.5 mm 3 months after the injection (Figure 3) and did not require a second injection even after 1 year.

### Case 2

A 58-year-old gentleman developed blepharoptosis with MRD1 of 0 mm and LMF of 4 mm for 7 months after surgery for a lacrimal gland tumor in the left eye. The blepharoptosis dramatically improved with MRD1 measured 4 mm immediately after FOOM flap shortening. Unfortunately, exposure keratopathy developed soon. Due to the progressive condition with a persistent corneal defect in spite of the frequent use of topical lubricants and autologous serum eye drops, medial and lateral tarsorrhaphy were performed. However, the sutures for tarsorrhaphy disrupted in 5 days due to the very powerful FOOM flap. Thus, we injected 10 units of botulinum toxin-A at the central supra-brow area in order to diminish its power. Four days thereafter, the upper eyelid excursion was reduced from 12 to 4 mm. He developed blepharoptosis and could close his left eye more completely. After that, the corneal defect gradually resolved. He also recovered from blepharoptosis with MRD1 of 3 mm after 3 months.

### Case 3

A 66-year-old gentleman presented with complete ptosis of the left eye after an operation for left frontal base meningioma 1 year ago. The MRD1 was −4 mm and LMF was 0 mm. The patient received FOOM flap shortening. Resolution of ptosis with MRD1 of 2 mm and upper eyelid excursion of 4 mm were achieved. However, after 1 month, exposure keratopathy developed despite the use of topical lubricants. We injected 10 units of botulinum toxin-A at the central supra-brow area. A week later, the MRD1 reduced to −1 mm with nearly no FOOM flap function. The exposure keratopathy gradually resolved. Four months after the injection, the MRD1 restored to 2 mm and stabilized. No second injection of botulinum toxin-A was needed.

## Discussion

There have been several reports regarding the utilization of either the advanced frontalis or orbicularis oculi muscle flap to lift the ptotic eyelid (13). Lai et al. first reported the FOOM flap shortening technique for blepharoptosis caused by various etiologies (10, 14). This technique provides several advantages, including a good operative field with no risk of intra-orbital neurovascular injury because the orbital septum is not opened (15, 16), which is an intraoperative challenge frequently encountered in secondary levator muscle repair. The situation may be even more complicated especially following an extensive orbital injury, including surgical trauma, which results in distorted orbital structure and severe cicatrization. In addition, the FOOM flap not only elevates the upper eyelid but also increases its excursion, which is hard to achieve with supramaximal levator resection or the frontalis sling operation.

Despite its many advantages, lagophthalmos with exposure keratopathy is a possible vision-threatening complication associated with this surgery. In most cases, surgeons may prescribe lubricants and normal eyelid closure may resume in 3-6 months; however, some patients develop irreversible corneal damage. Tarsorrhaphy to forcibly close the patient's eye is effective, but the cosmetic appearance is often unacceptable. Occasionally, the sutures for tarsorrhapy would disrupt because of the powerful FOOM flap. In severe cases, surgical revision may be needed to partially or totally release the attachment of FOOM flap, which will permanently decrease its function. In this case series, we demonstrated that the injection of botulinum toxin-A, a quick, minimal invasive procedure, may be a safe and effective alternative to manage this complication. Previous studies have shown that injections of botulinum toxin-A may treat upper eyelid retraction successfully (17). In this scenario, the goal is to reduce the LMF. On the contrary, FOOM flap is composed of the frontalis and the orbicularis oculi muscle, and the frontalis muscle provides the suspension power to elevate the eyelid through its connection with the orbicularis oculi muscle. Therefore, in patients developing exposure keratopathy after FOOM flap surgery, the most straightforward treatment is to decrease the power of the frontalis muscle. In our cases, we injected botulinum toxin-A at central supra-brow area around the origin of FOOM flap. Indeed, their FOOM flap functions were reduced after 4-7 days and recovered 3 months thereafter. Even though they developed blepharoptosis again after this procedure, the appearances were relatively acceptable when compared to those with tarsorrhaphy. Most importantly, the effect of botulinum toxin-A, in contrast to the surgical revision, was temporary and the FOOM flap would recover its elevation power with resolution of blepharoptosis. Therefore, we suggest that injections of botulinum toxin-A may be a promising method to treat exposure keratopathy following FOOM flap shortening without permanent effects on the function of the FOOM flap (Table 1).

**Table 1:** Comparison among different treatments for refractory exposure keratopathy after FOOM flap shortening.

	**Methods**		
**Characteristics**	**Revision**	**Tarsorrhaphy**	**Botulinum toxin-A injection**
Surgery	+	+	-
Procedure time	Hours	Minutes	Seconds
Onset of effect	Immediate	Immediate	4-7 days
Duration of effect	Permanent	Variant	3 months
Ptosis	+	Forcible eye closure	+
Cosmetic appearance	Acceptable	Unacceptable	Acceptable
Permanent effect on FOOM flap function	+	-	-

## Conclusions

In blepharoptosis with poor LMF related to orbital trauma including surgery, FOOM flap shortening is an alternative technique to achieve not only a satisfactory appearance but also good function of the upper eyelid, as in the three cases reported herein. Still, lagophthalmos with exposure keratopathy is a potential complication. Botulinum toxin-A injection might be a very effective treatment for patients developing this complication after FOOM flap surgery.

## Data Availability Statement

The raw data supporting the conclusions of this article will be made available by the authors, without undue reservation.

## Ethics Statement

Ethical review and approval was not required for the study on human participants in accordance with the local legislation and institutional requirements. Written informed consent was obtained from the individual(s) for the publication of any identifiable images or data included in this article.

## Author Contributions

C-CLai collected the clinical data and prepared the manuscript. Both authors reviewed and revised the manuscript.

## Funding

The publication fees are funded by the Department of Ophthalmology, National Cheng Kung University Hospital.

## Conflict of Interest

The authors declare that the research was conducted in the absence of any commercial or financial relationships that could be construed as a potential conflict of interest.

## Publisher's Note

All claims expressed in this article are solely those of the authors and do not necessarily represent those of their affiliated organizations, or those of the publisher, the editors and the reviewers. Any product that may be evaluated in this article, or claim that may be made by its manufacturer, is not guaranteed or endorsed by the publisher.
